# Incidental Finding of Inferior Vena Cava Atresia Presenting with Deep Venous Thrombosis following Physical Exertion

**DOI:** 10.1155/2015/146304

**Published:** 2015-11-10

**Authors:** Shalini Koppisetty, Alton G. Smith, Ravneet K. Dhillon

**Affiliations:** ^1^Department of Radiology, Beaumont Health System, Grosse Pointe, MI 48230, USA; ^2^Emergency Medicine, Henry Ford Health System, West Bloomfield, MI 48322, USA

## Abstract

Inferior vena cava atresia (IVCA) is a rare but well described vascular anomaly. It is a rare risk factor for deep venous thrombosis (DVT), found in approximately 5% of cases of unprovoked lower extremity (LE) DVT in patients <30 years of age. Affected population is in the early thirties, predominantly male, often with a history of major physical exertion and presents with extensive or bilateral DVTs. Patients with IVC anomalies usually develop compensatory circulation through the collateral veins with enlarged azygous/hemizygous veins. Despite the compensatory circulation, the venous drainage of the lower limbs is often insufficient leading to venous stasis and thrombosis. We describe a case of extensive and bilateral deep venous thrombosis following physical exertion in a thirty-six-year-old male patient with incidental finding of IVCA on imaging.

## 1. Introduction

Inferior vena cava atresia (IVCA) is an extremely rare vascular anomaly with an estimated prevalence of approximately 1% in general population [1]. It is also called IVC agenesis or aplasia. IVCA is found in approximately 5% of cases of unprovoked lower extremity (LE) deep venous thrombosis (DVT) in young adults, significantly occurring before the fourth decade of life [2].

## 2. Case Presentation

A previously healthy thirty-six-year-old male presented to the emergency department with acute onset of shortness of breath and diffuse bilateral lower extremity pain and swelling for two days. Patient was dancing for two hours in a party and the following day woke up with severe bilateral lower extremity and lumbar pain. The patient eventually developed shortness of breath and worsening pain in his legs and back. Past medical history and family history were insignificant.

### 2.1. Hospital Course

On arrival, the patient's vitals were as follows: blood pressure 130/100, pulse 88. Respiratory rate was 20, oxygen saturation 100% on room air. On physical examination there was bilateral lower extremity swelling, tenderness, tense skin, and blotchy purplish discoloration of both legs. Additional lab work including prothrombin time, basic metabolic profile, and complete blood picture was unremarkable. Venous Duplex scan of LE revealed extensive, bilateral DVT. Computer tomography (CT) scan of the chest was negative for pulmonary embolism.

### 2.2. Bilateral Venous Duplex Ultrasound Lower Extremity

Total occluding acute thrombosis of right external iliac vein, right and left common femoral vein, right and left femoral veins, right and left greater saphenous vein, right and left small saphenous vein, right and left popliteal vein, right and left gastrocnemius vein, left peroneal veins, right and left posterior tibial veins, and right and left soleal vein was observed.

### 2.3. CT Scan of the Chest, Abdomen, and Pelvis with Contrast

**Figure 1:**
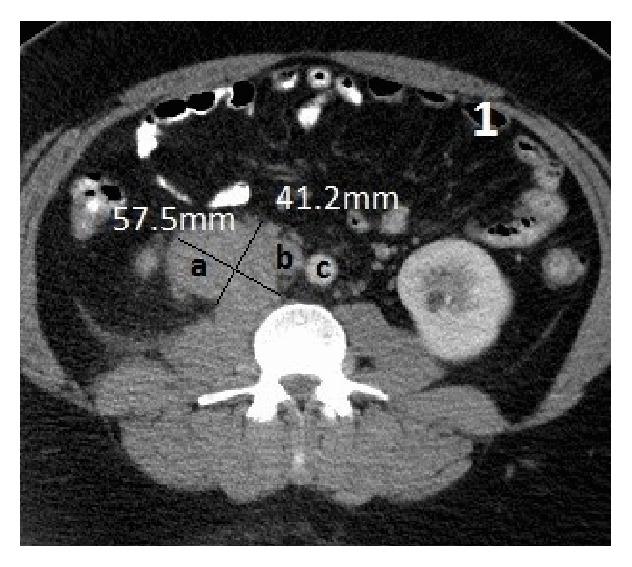
Abdomen CT axial view: (a) inferior vena cava (IVC) varix measuring 57.5 mm × 41.2 mm, (b) IVC, and (c) aorta.

**Figure 2:**
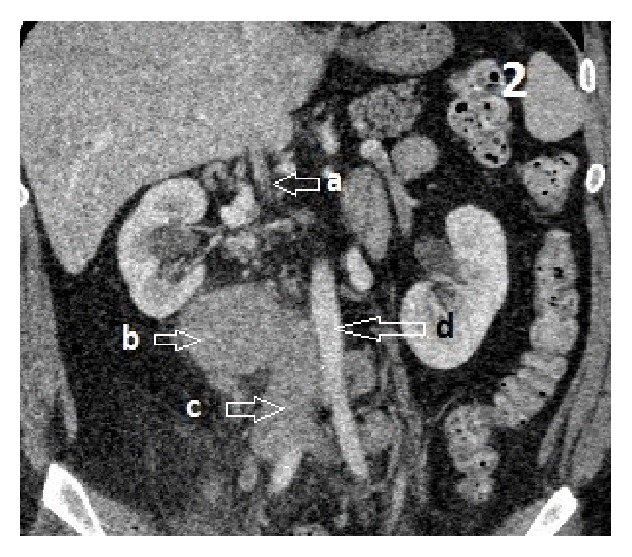
Abdomen CT coronal view: (a) atretic intrahepatic IVC, (b) IVC venous varix, (c) thrombosed IVC, and (d) aorta.

**Figure 3:**
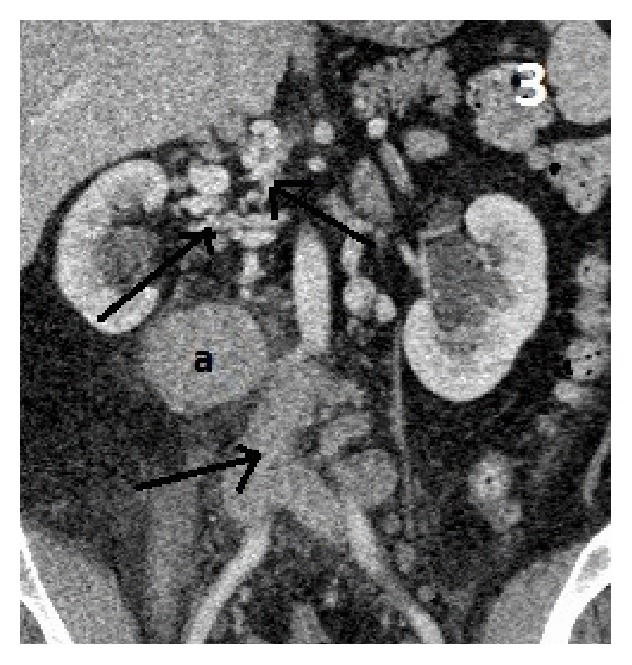
Abdomen CT coronal view: (a) IVC venous varix. Arrows pointing enlarged, multiple retroperitoneal/paralumbar collateral veins.

**Figure 4:**
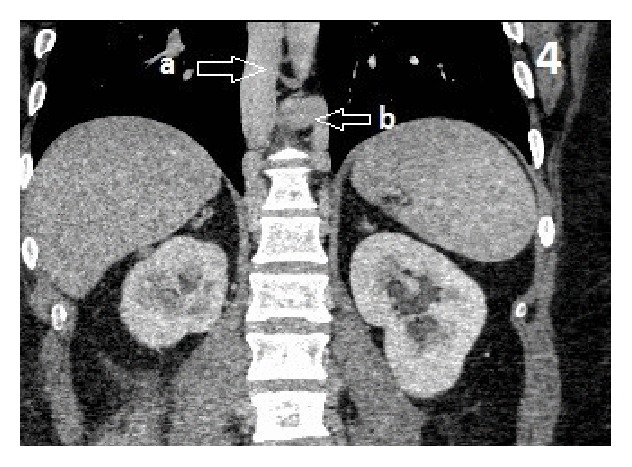
Abdomen CT coronal view: (a) enlarged azygous vein, (b) enlarged hemizygous vein.

Approximately 5.7 × 4.1 cm right retroperitoneal mass at the level of L3 adjacent to the IVC, differential, would be large venous varix or lymph node (Figure 1). Atretic intrahepatic portion of the IVC with multiple collaterals is seen in the retroperitoneum as well as a dilated hemiazygous and azygous system and response to the atretic intrahepatic IVC (Figures 2, 3, and 4).

### 2.4. Treatment

Patient was transferred to surgical unit for pharmacomechanical catheter-directed thrombolysis (PCDT). Patient underwent a venogram, ultrasound guided venous puncture bilateral popliteal veins, sheath placement, and placement of bilateral Ekos infusion catheters. He received continuous thrombolysis with tissue plasminogen activator (tPA) and heparin infusions. Subsequent venogram showed moderate improvement in bilateral venous flow. Balloon angioplasty was performed on the bilateral common iliac veins with subsequent sluggish venous flow. Mechanical thrombectomy of left and right iliac veins was then performed using Trellis device with infusion of tPA on both sides. Serial fibrinogen levels were monitored during the procedure. Final venogram showed patency of the iliac veins and lower IVC with persistent but improved sluggish flow. Patient was discharged home on oral Xarelto (rivaroxaban) and compression stockings.

### 2.5. Outcome and Follow-Up

Magnetic resonance imaging (MRI) of the abdomen with and without contrast performed after 2 weeks confirmed the retroperitoneal mass most likely related to large thrombosed venous varix. The IVC was patent and of normal caliber at and above the level of the hepatic veins. IVC above the level of the renal veins was occluded with multiple collaterals. The left renal vein was small in caliber but patent. There was a small amount of residual eccentric nonocclusive thrombus within the right external iliac vein. Patient was in regular follow-up for one year without any recurrent DVT.

### 2.6. Diagnosis

Diagnosis of the patient was as follows: (1) inferior vena cava atresia and (2) deep venous thrombosis, bilateral lower limbs.

## 3. Discussion

Inferior vena cava anomalies are rare and have an estimated prevalence of 0.07% to 8.7% in general population [2]. IVC atresia is a type of IVC anomaly and accounts for up to 5% of unprovoked DVTs in young patients aged 20–40 years. IVC atresia can be congenital or acquired and is thought to be due to embryonic dysgenesis or thrombosis during the intrauterine or perinatal period [2, 3]. During embryogenesis (6–8 weeks of gestation), the IVC is formed by the fusion of three sets of paired veins (posterior cardinal, subcardinal, and supracardinal veins) [4]. Failure of these paired veins to fuse into a unilateral right-sided venous structure results in anomaly of IVC. Patients with IVCA develop a robust collateral deep venous system with or without azygous and hemizygous continuation of the IVC. Lambert et al. propose that the collaterals are unable to cope with the demands of increasing blood flow, thereby generating venous stasis, ulceration, and recurrent DVT [5]. Most of the patients with IVCA are asymptomatic and detected incidentally during radiological procedures or abdominal surgery [6]. Common symptoms are lower extremity pain, swelling, ulcers, and sometimes nonspecific pain in the lower back and abdomen. Pulmonary embolism is not a frequent finding with IVCA, because the emboli get trapped in the azygous/hemizygous system preventing it from reaching the pulmonary circulation [5]. The characteristic findings of IVCA-DVT include occurrence before the fourth decade of life, male predominance, extensive or bilateral LE DVT, and history of major physical exertion [5, 7, 8]. The DVT most frequently involves the distal IVC, common, internal, and external iliac and femoral veins [8]. Data suggests that approximately one-third of patients with IVCA had associated hypercoagulability disorders [9]; hence screening is recommended for any coagulation abnormalities. IVC anomalies can cause diagnostic problems because of tumor like appearance of the enlarged azygous or collateral veins on imaging, which was indeed what happened in this case. Our patient had a nonspecific retroperitoneal mass at the level of L3 on CT scan of the abdomen, which was confirmed most likely as a venous varix of IVC on MRI. Although ultrasound and venography are excellent tools in identification of DVT, they can often miss the diagnosis of IVCA. CT or MRI studies are more effective in identifying IVC anomalies and are recommended if there is history of unprovoked DVT in younger patients. Review of the literature revealed that IVC anomalies may be associated with renal anomalies, with a handful of case reports reported in literature. Most of these cases had absent IVC with renal anomalies involving the right kidney (agenesis, hypoplasia, and aplasia). Gayer et al. reported the occurrence of congenital anomalies of the IVC and right renal aplasia in three patients detected on CT scan. The authors proposed that the association between absent IVC and right renal anomalies was not coincidental and may be due to abnormal embryogenesis [10]. Absent IVC can lead to impaired venous drainage of the right metanephros, resulting in right kidney abnormalities. The left metanephros drain via gonadal vein and lumbar perforators, leading to less common involvement of the left kidney. However there are few reported cases of absent IVC with left renal hypoplasia and atrophy [11–13]. Van Veen et al. suggested the term KILT syndrome, due to the occurrence of kidney and IVC abnormalities associated with leg thrombosis [11]. There are no standard guidelines for the treatment of IVCA-DVT and further research is required on optimal treatment strategies. Patients are initially treated with intravenous heparin followed by oral anticoagulation. Recently, Broholm et al. described the efficacy of catheter directed thrombolysis (CDT) for rapid thrombus removal in patients with IVCA with acute DVT, especially involving iliofemoral venous thrombosis [14]. Further the authors proposed that CDT provides immediate symptom relief and significantly decrease thrombus burden that may otherwise takes days to weeks to resolve with systemic anticoagulation alone [14]. In patients with extensive iliofemoral DVT, pharmacomechanical catheter directed thrombolysis (PCDT), which refers to the combination of mechanical thrombectomy and CDT, has been shown to significantly decrease the thrombus burden, incidence of recurrent DVT, and incidence of post thrombotic syndrome compared to systemic anticoagulation alone [15]. Observational studies demonstrated promising results with PCDT; however more research trials are necessary to look into long term efficacy [16–19]. Patients are strongly advised to wear elastic stocking support and leg elevation and also avoid risk factors such as excessive physical exertion, prolonged immobilization, smoking, and hormonal contraceptive use [5, 8]. Long term anticoagulation may be required if associated with hereditary thrombophilia and other risk factors due to risk of recurrent thrombosis.

## 4. Conclusion

It is essential for physicians to consider the possibility of IVC anomalies in a young adult presenting with unexplained, extensive, or bilateral LE DVT. The diagnosis can be challenging and requires detailed imaging studies with computed tomography and magnetic resonance imaging to identify IVC anomalies. Further diagnostic workup and management should be considered for any coagulation abnormalities and long term anticoagulation. Pharmacomechanical catheter-directed thrombolysis followed by systemic anticoagulation seems a promising therapy with significant reduction of thrombus burden.
